# Ghostformer: A GhostNet-Based Two-Stage Transformer for Small Object Detection

**DOI:** 10.3390/s22186939

**Published:** 2022-09-14

**Authors:** Sijia Li, Furkat Sultonov, Jamshid Tursunboev, Jun-Hyun Park, Sangseok Yun, Jae-Mo Kang

**Affiliations:** 1 Department of Artificial Intelligence, Kyungpook National University, Daegu 41566, Korea; 2 Department of Information and Communications Engineering, Pukyong National University, Busan 48513, Korea

**Keywords:** small object detection, GhostNet, *regional proposals*, two-stage transformer

## Abstract

In this paper, we propose a novel two-stage transformer with GhostNet, which improves the performance of the small object detection task. Specifically, based on the original Deformable Transformers for End-to-End Object Detection (deformable DETR), we chose GhostNet as the backbone to extract features, since it is better suited for an efficient feature extraction. Furthermore, at the target detection stage, we selected the 300 best bounding box results as *regional proposals,* which were subsequently set as primary object queries of the decoder layer. Finally, in the decoder layer, we optimized and modified the queries to increase the target accuracy. In order to validate the performance of the proposed model, we adopted a widely used COCO 2017 dataset. Extensive experiments demonstrated that the proposed scheme yielded a higher average precision (AP) score in detecting small objects than the existing deformable DETR model.

## 1. Introduction

In the realm of natural language processing (NLP), the transformer model has become the preferred solution to tackle tasks such as question answering, text classification, machine translation, and document summary [1,2,3,4,5]. The ability of transformer modules to learn complex relationships between input sequences via self-attention is critical for its success [6,7]. Inspired by the success of the transformer in the NLP research area, Dosovitskiy et al. proposed The Vision Transformer (ViT) to tackle the image recognition task in the image domain [8]. Recently, the pyramid vision transformer (PVT), which overcame the difficulties of porting tranformer to various dense prediction tasks, was proposed [9]. In addition, the authors in [10] proposed a texture transformer network for image super-resolution (TTSR), which was the first scheme to utilize transformer architecture for image generation tasks. However, transformers that effectively utilize low-level image features require further investigation [11].

Current object detection pipelines, such as End-to-End Object Detection with Transformers (DETR) and Deformable Transformers for End-to-End Object Detection (deformable DETR) combine transformers with a backbone model based on convolutional neural networks (CNNs) to extract features; then, they exploit the modified encoder–decoder architecture of the transformer module to categorize and detect objects within an image [12,13,14,15].

Most of the current state-of-the-art models based on transformers tend to use ResNet50 as a backbone network, which was proposed by He et al. to address the vanishing gradient problem through the residual connections that enable designing deep networks. Despite its good performance in image recognition tasks, it requires a long time to converge, due to its model size. The GhostNet model is proposed to replace the computationally heavy standard convolution operation with a more efficient Ghost module. More precisely, the Ghost module initially generates some part of the total output feature map, while the rest of the feature maps are generated through a cheap linear operation, i.e., depthwise separable convolutions. Finally, the feature maps generated through two stages are concatenated [16,17,18]. In comparison to standard CNNs, GhostNet’s total number of parameters and computational complexity are reduced without affecting the size of the output feature maps.

The function of the transformer models is to calculate the spatial position of the input image. The goal of the encoder is to determine the features of the image, and the object detection is performed by the decoder [13]. In the image domain, object queries in the decoder are not related to the current image, so the accuracy rate is low. Here, we adopt *regional proposals* to ensure object queries have a stronger relationship with an image [13]. Traditional end-to-end convolutional models and transformers with CNN based feature extractors perform well on tasks that include the recognition and detection of large objects [13,19,20]. However, the previously proposed transformer models struggle to yield desirable results in the presence of small objects within an image [12].

We aim to address this critical issue by proposing a novel two-stage transformer that is robust to detecting small objects. Our proposed method increased small-object detection accuracy through a more efficient network, namely GhostNet, which generates more features using fewer parameters. Furthermore, we added one more stage after the encoder that generated regional proposals to refine object queries before they entered the decoder layer [21]. Extensive experiments demonstrated that our proposed model yielded superior results in the detection of small objects compared to deformable DETR.

The remainder of this paper is organized as follows: Section 2 discusses related works. Section 3 and Section 4 present the proposed approach and the experimental results, respectively. Finally, Section 5 concludes this paper.

## 2. Related Work

Most object detection models based on CNNs exploit several hand-crafted techniques such as anchor generation and non-maximum suppression (NMS) postprocessing methods to improve performance [21,22,23]. DETR proposed by Carion et al. effectively eliminates the need for such techniques; thus, it is considered the first end-to-end object detector in the image domain [12]. The architecture of the DETR includes a backbone network based on CNNs that generate feature maps and a transformer encoder–decoder model that accepts the output feature maps of the backbone for further processing. Despite its end-to-end nature and competitive performance, DETR contains some critical issues that need to be addressed. Specifically, it exhibits relatively slower convergence than modern CNN-based object detectors due to the difficulty in training attention models that process the image features. Furthermore, DETR suffers from inferior performance in small object detection task compared to the most modern object detectors. These utilize high-resolution feature maps that result in better detection of small objects. However, high-resolution feature maps lead to an unacceptable complexity for DETR, as the encoder part of DETR has a quadratic complexity with the spatial size of input feature maps. In order to address the abovementioned issues of DETR, Zhu et al. proposed a deformable DETR that achieved better results in detecting small objects and also converged faster than DETR [13]. Inspired from deformable convolutions, the authors introduced a *deformable attention module* that focused only on a small set of key sampling points in the neighborhood that were not fixed in position but learnable (as deformable convolution), enabling a local and sparse efficient mechanism. The slow convergence issue can be alleviated by attending only to a small portion of keys for each query.

The deformable attention module is calculated as follows:

(1) $$\begin{array}{cc} \; & DeformAttn(z_{q},t_{q},x)=\\ \; & \sum_{m=1}^{M}W_{m}[\sum_{k=1}^{K}A_{mqk}W_{m}^{{}^{\prime }}x\left(t_{q}+\Delta t_{mqk}\right)] \end{array}$$

where *q* and *k* represent query and key, respectively. $z_{q}$ stands for a query generated by *x* through linear transformation. $t_{q}$ indexes a 2-D reference point, and $\Delta t_{mqk}$ represents the location offset of the sampling collection point with respect to the reference points, named offsets, which is obtained from the query feature through the fully connected layer.

Furthermore, *m* denotes attention head, and $W_{m}$ is the result of a linear transformation of attention after the value is applied to the output of different heads. $W_{k}$ is used to transform $x_{k}$ into value. $A_{mqk}$ represents a normalized weight of attention. In fact, each query samples *K* positions in each head, only interacting with the features of those locations, and $x(t_{q}+\Delta t_{mqk})$ represents the value interpolated based on the location of the sampling point.

In the encoder part of the deformable DETR, the transformer attention modules are substituted for the proposed multiscale deformable attention module. Specifically, the encoder of the model accepts a multiscale feature map and generates another multiscale feature map with the same resolution size as the input. The input feature maps of the encoder are extracted from the output feature maps of stages $C_{3}$ to $C_{5}$ (transformed by a 1×1 convolution), where $C_{l}$ is of resolution $2^{l}$ lower than the input image. The lowest resolution feature map $x^{L}$ is obtained via 3×3 convolution with stride 2 on the final $C_{5}$ stage, denoted as $C_{6}$. The channel sizes of multiscale feature maps are equal to 256. Finally, in addition to the positional embedding, scale-level embeddings are adopted in order to identify the location of query pixels.

Combining deformable attention with multiscale features, the multiscale deformable attention module is calculated as follows:

(2) $$\begin{array}{cc} \; & MSDeformAttn(z_{q},t_{q},{\left(x^{l}\right)}_{l=1}^{L})=\\ \; & \sum_{m=1}^{M}W_{m}[\sum_{k=1}^{K}\sum_{l=1}^{L}A_{mqk}W_{m}^{{}^{\prime }}x^{l}\left(\phi_{l}\left({\hat{t}}_{q}\right)+\Delta t_{mqk}\right)] \end{array}$$

where *m* indicates the attention head, *l* stands for the input feature level, and *k* represents the sampling point. ${\left(x^{l}\right)}_{l=1}^{L}$, here, we set L=4. The normalized coordinates ${\hat{t}}_{q}$ are rescaled to the input feature map of the *l*-th level through function $\phi_{l}\left({\hat{t}}_{q}\right)$, so that each reference point has a corresponding (normalized) coordinate at all feature layers to facilitate the calculation of the locations of those points sampled at different feature layers. The rest of Equation (2) is processed consistent with the deformable attention module formula in (1).

The decoder of the DETR consists of self-attention and cross-attention modules. However, the authors of the deformable DETR propose to replace the cross-attention module with the introduced multiscale attention module, as their model is designed for processing convolutional feature maps as key elements. For each object query, the 2-dimensional (2-D) normalized coordinate of the reference point ${\hat{t}}_{q}$ is predicted from its object query embedding via a learnable linear projection followed by a sigmoid function.

## 3. Proposed Approach

Although the capability of ResNet has been proven in many applications, it has a significant drawback in that the deep network usually requires several weeks of training time. As a result, the cost of training is very high. In addition, object queries in the decoder of the deformable DETR are not correlated to the image. In order to mitigate these shortcomings effectively, we selected GhostNet as the feature extractor, and we employed regional proposals to feed object queries into the decoder and refined the object queries six times during the decoding.

### 3.1. GhostNet

**Figure 1:**
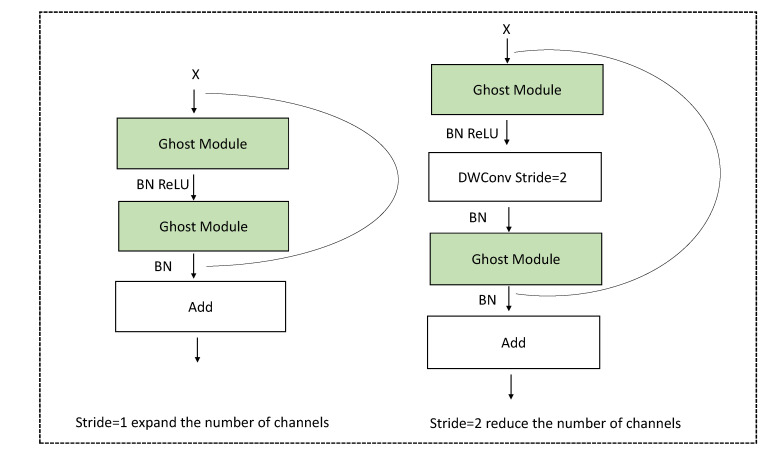
Ghost bottleneck.

The GhostNet generates more feature maps with fewer parameters, which makes it a better option for extracting small target feature maps. In this paper, due to its efficiency, we adopted GhostNet as a backbone network instead of the traditional ResNet. GhostNet is mostly made up of Ghost bottlenecks, with Ghost modules serving as the building blocks. The first layer is a typical convolutional layer with 16 filters, followed by a sequence of Ghost bottlenecks with increasing channel counts. These Ghost bottlenecks are classified into stages based on the sizes of their input feature maps. The Ghost bottleneck can be considered as the basic residual block in ResNet, in which several convolutional layers and shortcuts are integrated. The Ghost bottleneck, as shown in Figure 1, is mostly made up of two stacked Ghost modules.

The first Ghost module’s task is to operate as an expansion layer, increasing the number of output and input channels as the expansion ratio increases. To match the shortcut path, the second Ghost module reduces the number of channels. Batch normalization and ReLU nonlinearity operations are performed after each layer, except that ReLU is not used after the second Ghost module [24,25]. Further, the shortcuts between the input and output of the two Ghost modules are concatenated. All Ghost bottlenecks are applied with a stride rate set to 1, except the last one of each stage at which the stride rate is set to 2. Finally, the global average pooling and convolutional layer are used to convert the feature map into the final 1280-dimensional feature vector. The computational cost of the Ghost module is significantly lower than the direct usage of conventional convolution.

**Figure 2:**
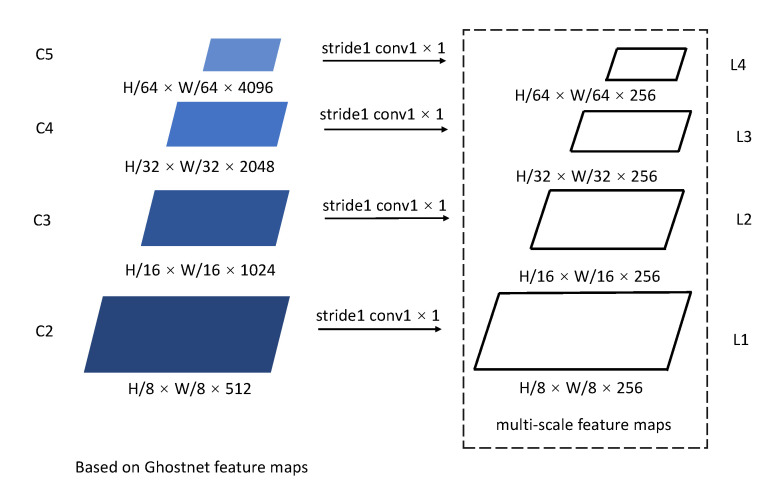
Generation of multiscale feature maps.

In order to generate multiscale feature maps, which were used as an input to the encoder layer, we adopted GhostNet as a backbone network. Unlike ResNet, in GhostNet, we extracted four layers of feature maps from $C_{2}$ to $C_{5}$, and the downsampling rates were set to 8, 16, 32, and 64, respectively. The resolution of the multiscale feature maps’ input and output was the same as in the encoder layer. In addition, the number of channels in all multiscale feature maps was set to 256 (see Figure 2).

### 3.2. Two-Stage Transformer

**Figure 3:**
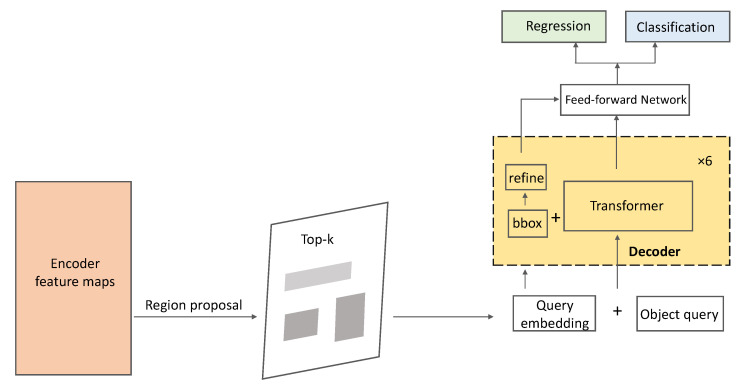
The details of the two-stage transformer with GhostNet in an encoder and a decoder layer.

The proposed scheme had a two-stage transformer (see Figure 3) process: (a) generating region proposals at the end of the encoder layer and (b) fine-tuning object queries several times in the decoder layer.

#### 3.2.1. The First Stage

Compared to the original deformable DETR, we removed the primary decoder and formed an encoder-only deformable DETR’s candidate area. Since our goal was to improve the correlation between the encoder and the object query in decoder, 300 top-scoring bounding boxes were selected as the regional proposals. In other words, the encoder layer output an object query for each pixel in the regional proposal, predicted a bounding box directly, and selected the highest scoring 300 bounding boxes as region proposals to be transferred to the next stage. In the encoder, the multiscale feature maps were mapped to a channel size of 256 dimensions in each layer. We set *i* as a pixel from feature level $l_{i}\in 1,2,\ldots ,L$ and 2-D normalized coordinates ${\hat{t}}_{i}=\left(t_{ix},t_{iy}\right)$. The bounding box $\Delta b_{i\left(x,y,w,h\right)}\in R$ was predicted by the branch of the bounding box regression as follows:

$$\begin{array}{c} {\hat{b}}_{i}=\sigma \left(\Delta b_{ix}+\sigma^{-1}\left({\hat{t}}_{ix}\right)\right),\sigma \left(\Delta b_{iy}+\sigma^{-1}\left({\hat{t}}_{iy}\right)\right),\\ \sigma \left(\Delta b_{iw}+\sigma^{-1}\left(s2^{l_{l-1}}\right)\right),\;\sigma \left(\Delta b_{iw}+\sigma^{-1}\left(s2^{l_{l-1}}\right)\right) \end{array}$$

where *s* is the target ratio, since the task was focused on small object detection. During the first stage, we generated different scales of bounding boxes starting from the earlier stages of feature maps to capture more fine-grained region proposals of small objects. However, the increased number of proposed bounding boxes led to the following issues: (a) the number of positive and negative samples were not evenly distributed, and (b) matching all of the assigned proposals to the ground truth resulted in slower convergence At the next stage, we addressed these issues.

#### 3.2.2. The Second Stage

In contrast to the deformable DETR, which employs self-attention modules and cross-attention techniques in the decoder part, we simply used the transformer itself as self-attention modules, as we changed the object query before entering the decoder. Additionally, we did not consider the reference point as the initial guess of the box center. Since the multiscale deformable attention module extracts image features around the reference point, offsets can be used to represent the difference between the predicted bounding box and the reference point.

We set the reference point, which is the output of the encoder, as the initial box center ${\hat{t}}_{i}=\left(t_{ix},t_{iy}\right)$; then, we normalized it using the sigmoid function and the inverse sigmoid function, denoted by σ(·) and $\sigma^{-1}\left(\cdot \right)$, respectively. The parameterizations of the four coordinates are as follows:

$$\begin{array}{c} \sigma \left(b_{ix}+\sigma^{-1}\left({\hat{t}}_{ix}\right)\right),\sigma \left(b_{iy}+\sigma^{-1}\left({\hat{t}}_{iy}\right)\right),\sigma \left(b_{iw}\right),\sigma \left(b_{ih}\right). \end{array}$$

Then, we used a forward iterative bounding box correction mechanism to improve the detection performance. We considered D=6 decoder layers, where the predicted layer was the (d−1)-th decoder layer; the coordinates of *d*-th decoder layer’s refined box are as follows:

$$\begin{array}{c} {\hat{b}}_{z}^{d}=\sigma \left(\Delta b_{zx}^{d}+\sigma^{-1}\left({\hat{b}}_{zx}^{d-1}\right)\right),\;\sigma \left(\Delta b_{zy}^{d}+\sigma^{-1}\left({\hat{b}}_{zy}^{d-1}\right)\right),\\ \sigma \left(\Delta b_{zw}^{d}+\sigma^{-1}\left({\hat{b}}_{zw}^{d-1}\right)\right),\;\sigma \left(\Delta b_{zh}^{d}+\sigma^{-1}\left({\hat{b}}_{zh}^{d-1}\right)\right) \end{array}$$

where d∈(1,2,…,D), σ(·) and $\sigma^{-1}\left(\cdot \right)$ are the sigmoid function and the inverse sigmoid function, respectively, and *D* stands for the number of decoder layers. $\Delta b_{z\left(x,y,w,h\right)}\in R$ are predicted by the branch of bounding box regression in different decoder layers.

## 4. Experimental Results

We conducted all the experiments using the COCO 2017 dataset [26]. The standard mean average precision (AP) metric was used to report results under different IoU (Intersection over Union) thresholds. The model was trained on the training set and evaluated on the validation set. There is a considerable number of small objects in the COCO 2017 dataset. Therefore, we reported the AP for comparison, especially $AP_{s}$, $AP_{m}$, and $AP_{l}$, which stand for the average precision of small objects, medium objects, and large objects, respectively. Specifically, small objects refer to the objects smaller than 32 × 32 pixels or objects which cover less than only 10% of the image, medium objects refer to the objects between 32 × 32 pixels and 96 × 96 pixels, and large objects refer to those more than 96 × 96 pixels.

Following the deformable DETR, we set *M* as the head of the multiscale deformable attention, which was equal to 8, and K=4 represented the index of the sample keys, which was the same as in the deformable attention. The parameters of the deformable transformer encoder were shared among different feature levels. By default, we used the Adam optimizer with a base learning rate set to $2\times 20^{-4}$, $\beta_{1}=0.9$, $\beta_{2}=0.999$, and weight decay equal to $10^{-4}$ [27]. All models were trained for 50 and 100 epochs. The learning rates of the fully connected layers that were used for predicting object query reference points and sampling offsets were multiplied by 0.1.

We first demonstrated the compatibility of a one-stage transformer (without the proposed method) with different backbones. As shown in Table 1, we evaluated the performance of the model equipped with ResNet-50 and GhostNet backbones, keeping the same training settings for both models. The experiments revealed that the model withthe GhostNet backbone took 85 h and showed 24.8%, 45.6%, 56.88% in the $AP_{s}$, $AP_{m}$, $AP_{l}$, respectively, improving from the original DETR model, which exploited ResNet-50 as a backbone network; meanwhile, the time was reduced by 7 h.

Furthermore, the regional proposal generation stage led to a boost in performance across all sizes when using the GhostNet as a backbone network, with the highest improvement of 3.5% detected in small objects compared to the conventional method. Increasing the number of epochs from 50 to 100 resulted in 28.9% $AP_{s}$, which was 1% better than that in the case of 50 epochs of training.

**Figure 4:**
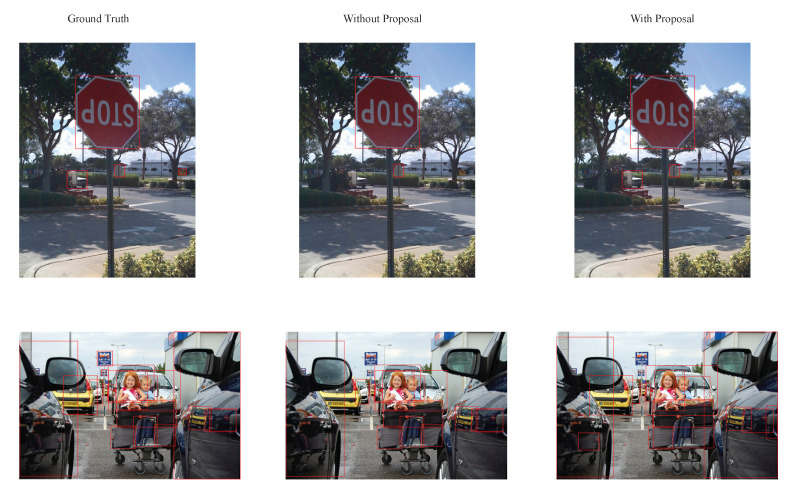
Comparison of visual results.

Figure 4 highlights the visual results of the original deformable DETR and the proposed two-stage transformer on two randomly chosen images from the validation set. As is evident from the Figure 4, the proposed model was able to detect more small objects than the deformable DETR.

As mentioned in the above sections, the number of positive and negative samples was not balanced for small object detection. We set different sample ratios between positive and negative under the same epoch according to the experimental experience of the previous two groups. To carry out our testing, we chose 100 epochs and the GhostNet as backbone.

Table 2 highlights that our proposed method led to improved results for small and medium targets by 0.3% and 0.1%, respectively. However, the AP value for the large objects was not affected by altering the sample locations.

Overall, when all the proposed techniques were included, the final experimental results for small object detection were 4.4% higher than the existing methods.

## 5. Conclusions

In this paper, we proposed a two-stage transformer combined with a GhostNet backbone network for the small object detection task. In order to capture more small objects, we extracted four layers of feature maps from the GhostNet backbone. Furthermore, in order to improve the performance, we first generated regional proposals at the end of the encoder layer and then finetuned the object queries in the decoder layer. The proposed method achieved superior results in terms of the average precision of small objects compared to the deformable DETR on the challenging COCO 2017 dataset. As a future research topic, we will study a lightweight small object detection scheme by accelerating the training process and evaluate it on practical application scenarios, e.g., risk detection for airport runways. 

## Figures and Tables

**Table 1:** Performance comparison of the proposed two-stage and conventional one-stage methods with Resnet-50 and GhostNet as a backbone.

Method	Times	Backbone	Epochs	${\mathrm{AP}}_{s}$	${\mathrm{AP}}_{m}$	${\mathrm{AP}}_{l}$
One-stage	92 h	ResNet-50	50	24.5	45.4	56.5
One-stage	85 h	GhostNet	50	24.8	45.6	56.8
Two-stage	96 h	ResNet-50	50	27.9	48.6	60.5
Two-stage	89 h	GhostNet	50	28.3	49.1	61.0
Two-stage	173 h	GhostNet	100	28.9	49.3	61.2

**Table 2:** Comparison with samples ratio.

Method	Samples Ratio	Epochs	${\mathrm{AP}}_{s}$	${\mathrm{AP}}_{m}$	${\mathrm{AP}}_{l}$
Two-stage	0.5	100	28.9	49.3	61.2
Two-stage	0.7	100	29.2	49.4	61.2

## Data Availability

Not applicable.
